# Role of Substrate in Au Nanoparticle Decoration by Electroless Deposition

**DOI:** 10.3390/nano10112180

**Published:** 2020-11-01

**Authors:** Luca Bruno, Mario Urso, Yosi Shacham-Diamand, Francesco Priolo, Salvo Mirabella

**Affiliations:** 1 Dipartimento di Fisica e Astronomia “Ettore Majorana”, Università di Catania, and IMM-CNR, via S. Sofia 64, 95123 Catania, Italy; mario.urso@dfa.unict.it (M.U.); francesco.priolo@ct.infn.it (F.P.); salvo.mirabella@dfa.unict.it (S.M.); 2 Department of Physical Electronics, School of Electrical Engineering and Department of Materials Science and Engineering, Faculty of Engineering, Tel Aviv University, Tel-Aviv 69978, Israel; YosiSh@tauex.tau.ac.il

**Keywords:** gold electroless deposition, nickel oxide nanostructures, decoration, nucleation model, growth, substrate effect

## Abstract

Decoration of nanostructures is a promising way of improving performances of nanomaterials. In particular, decoration with Au nanoparticles is considerably efficient in sensing and catalysis applications. Here, the mechanism of decoration with Au nanoparticles by means of low-cost electroless deposition (ELD) is investigated on different substrates, demonstrating largely different outcomes. ELD solution with Au potassium cyanide and sodium hypophosphite, at constant temperature (80 °C) and pH (7.5), is used to decorate by immersion metal (Ni) or semiconductor (Si, NiO) substrates, as well as NiO nanowalls. All substrates were pre-treated with a hydrazine hydrate bath. Scanning electron microscopy and Rutherford backscattering spectrometry were used to quantitatively analyze the amount, shape and size of deposited Au. Au nanoparticle decoration by ELD is greatly affected by the substrates, leading to a fast film deposition onto metallic substrate, or to a slow cluster (50–200 nm sized) formation on semiconducting substrate. Size and density of resulting Au clusters strongly depend on substrate material and morphology. Au ELD is shown to proceed through a galvanic displacement on Ni substrate, and it can be modeled with a local cell mechanism widely affected by the substrate conductivity at surface. These data are presented and discussed, allowing for cheap and reproducible Au nanoparticle decoration on several substrates.

## 1. Introduction

Metal decoration of nanostructures is emerging as a promising methodology for adding functionalities and developing materials properties, with applications to electronics, sensing, catalysis, and computing industries [1,2,3,4,5,6,7]. Nanostructures covered with metal nanoparticles interest the scientific community also for basic understanding in synthesis and functional properties. Au-decorated nanostructures are shown to be excellent performing in sensor devices, such as electrochemical [8] or biochemical [9] sensors and optical [10] and gas [11] detectors. Among the different nanostructures decorated with gold, Ni-based nanostructures have attracted extensive interests because of their novel optical, electronic, electrochemical, magnetic, thermal, and mechanical properties [12] and potential application in a great variety of fields, such as energy storage and sensing [13,14,15]. The decoration of nanostructures can be achieved through solution methods, allowing in principle scalability and cost-effectiveness. Still, a deeper understanding and a careful protocol are needed to ensure repeatability and effective decoration.

Au electroless deposition (ELD) [16] represents an attractive solution-based method for decorating nanostructures, allowing low cost and versatility for depositing films or nanoparticles [17,18,19,20,21,22,23,24]. A reliable method for Au cluster decoration could be used in several applications such as DNA sensing [25], UV sensing [26], and gas sensing [27].

Despite the large number of experimental works, there is no clear consensus on the gold deposition mechanism, which is shown to be affected by bath pH and stability, reducing agents, temperature, and gold ion source [28]. In Au ELD, the most common ion source is KAu(CN)_2_, despite the fact that the accumulation of free cyanide ions (causing plating rate reduction) sometimes moved the choice towards non-cyanide bath using trivalent Au salts (KAuCl_4_, HAuCl_4_, KAuO_2_, KAu(OH)_4_). Nevertheless, the use of non-cyanide bath determines a too-fast Au deposition [18,19,29,30], which is non-optimal for a manageable coverage of nanostructures [18,22]. A low and constant deposition rate is a prerequisite to obtaining a reproducible Au nanoparticle decoration, and KAu(CN)_2_ as a gold ion source seems to be the best choice. In addition, a reducing agent for the metal ions is typically present in the ELD bath, influencing the kinetics of electroless deposition as well as surface morphology and physicochemical properties of the deposits [19]. The most used reducing agent is sodium hypophosphite [28], whose effect on the Au deposition rate has been shown by Vorobyova et al. [28] to be a catalytic action through oxidation of substrate.

The electroless gold plating on nickel is shown to proceed by both mechanisms of cementation and catalytic gold reduction by hypophosphite [31]. According to Vorobyova, et al., the gold substitution by nickel from the substrate occurs simultaneously with the gold reduction by hypophosphite [31]. Such a process could be highly damaging for Ni nanostructures, as the Ni displacement would irreversibly modify or destroy the nanostructures to be decorated. In this work we investigated the Au decoration of Ni-based nanostructures by means of the ELD process. In particular, the effect of substrate is quantitatively investigated, using an ELD bath of KAu(CN)_2_ and Na hypophosphite. In addition, substrates are preventively treated with hydrazine hydrate to induce a cooperative reducing action with hypophosphite. By a comparison with several substrates (flat or nanostructured, metal or semiconductor) a general model for the Au ELD mechanism is proposed.

## 2. Materials and Methods

### 2.1. Synthesis

We used 5 different substrates that were obtained as follows: Ni substrate is a Ni film (100 nm thick) evaporated (Kenosistec evaporator, Binasco, MI, Italy, $6\times 10^{-7}$ mbar pressure) on crystalline-Si (*c*-Si) samples; NiO substrate (200 nm thick) was obtained by annealing (at 500 °C, 3 h in O_2_ atmosphere) the Ni substrate; *n*- and *p*-type Si substrates were cut Czochralski (Cz) wafers (resistivity of 0.001–0.01 Ω cm); NiO nanowalls (NWLs) substrate was obtained by chemical bath deposition (CBD) by immersing the Ni substrate in solution of 0.42 M NiSO_4_ · 6H_2_O (Alfa Aesar, Kandel, Germany, 98%), 0.07 M K_2_S_2_O_8_ (Alfa Aesar, Kandel, Germany 97%) and 3.5 wt% ammonia (Merck, Darmstadt, Germany, 30–33 wt% NH_3_ in H_2_O) for 20 min at 50 °C, and by thermal annealing at 350 °C in vacuum for 1 h [15,32].

All substrate underwent the same preparation. After rinsing with deionized water and drying in N_2_ gas flow, substrates were immersed in 7.5 *v/v%* hydrazine hydrated (HH, N_2_H_4_ in H_2_O, Sigma-Aldrich, St. Lous, MO, USA, 50–60%) for 10 min at room temperature in order to activate their surface [33,34]. The HH step is intended as a reducing process for the surface prior to its immersion in the ELD bath, with the aim to provide more nucleation sites for Au deposition.

The solution for electroless deposition (ELD) consisted of 70 g/L NH_4_Cl (Sigma-Aldrich, St. Lous, MO, USA, ≥99.5%), 45 g/L Na_3_C_6_H_5_O_7_ · H_2_O (Sigma-Aldrich St. Lous, MO, USA), 2 g/L KAu(CN)_2_ (Sigma-Aldrich, St. Lous, MO, USA, 98%) and 8 g/L NaH_2_PO_2_ (Sigma-Aldrich, St. Lous, MO, USA, ≥99%). Bath pH was adjusted to 7.5 by using a 0.1 M NaOH solution. After HH bath, samples were subsequently immersed in ELD solution, held at 80–85 °C through a bain-marie configuration, for different durations (Table 1). ELD bath pH and temperature were constantly monitored. After ELD, the Au-decorated samples were rinsed with deionized water to remove unwanted precipitates and dried in N_2_ gas flow. ELD samples are labelled using substrate and ELD immersion time (e.g., Ni_90 means a Ni substrate immersed for 90 s in ELD solution).

### 2.2. Characterization

Surface morphology and thickness of samples were characterized by using a scanning electron microscope (Gemini field emission SEM Carl Zeiss SUPRA 25, FEG-SEM, Carl Zeiss Microscopy GmbH, Jena, Germany) combined with energy dispersive X-ray spectroscopy (EDX). SEM images were analyzed by using ImageJ software [35]. Rutherford backscattering spectrometry (RBS, 2.0 MeV He^+^ beam at normal incidence) with a 165° backscattering angle was performed by using a 3.5 MV HVEE Singletron accelerator system (High Voltage Engineering Europa, Netherlands). RBS spectra were analyzed by using XRump software [36], allowing us to determine the amount of Ni and Au.

## 3. Results

**Figure 1:**
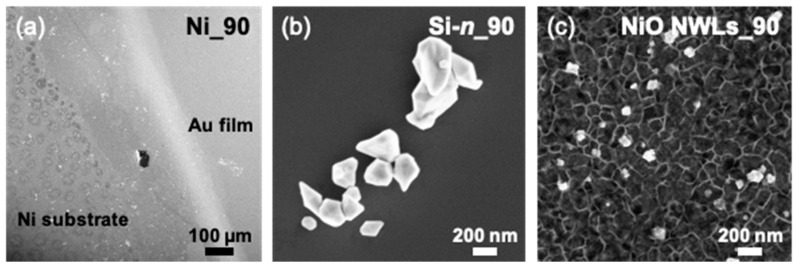
Plan-view SEM images of (**a**) Ni, (**b**) Si-*n*, and (**c**) NiO nanowalls (NWLs) samples obtained by Au ELD for 90 s. Note that the marker in (**a**) is considerably larger than elsewhere.

Figure 1 reports the plan-view SEM images of Au-decorated samples obtained after immersion in ELD solution for 90 s. Ni_90 (Figure 1a), Si-*n*_90 (Figure 1b) and NiO NWL_90 samples (Figure 1c) show Au deposits with dramatically different morphologies between them. An uniform Au film can be seen at this low magnification in the Ni_90 sample (the Au presence is ensured by EDX spectrum, Figure S1), while the presence of clusters with irregular morphology is assessed for other substrates: Si-*n*_90, Si-*p*_90 (Figure S2) and NiO NWLs_90. The expected grain size for the Ni substrate [33] is not visible at this magnification. Furthermore, it should be noted that Figure 1b,c are not representative of the whole sample, as these clusters have a quite low density. We observe that Au clusters on NiO NWLs_90 are considerably smaller than those on Si. The substrate plays a paramount role in the ELD of Au, driving the Au deposition from a film overlayer (Ni substrate) to a cluster decoration (Si, NiO NWLs substrates) with a different mean size.

**Figure 2:**
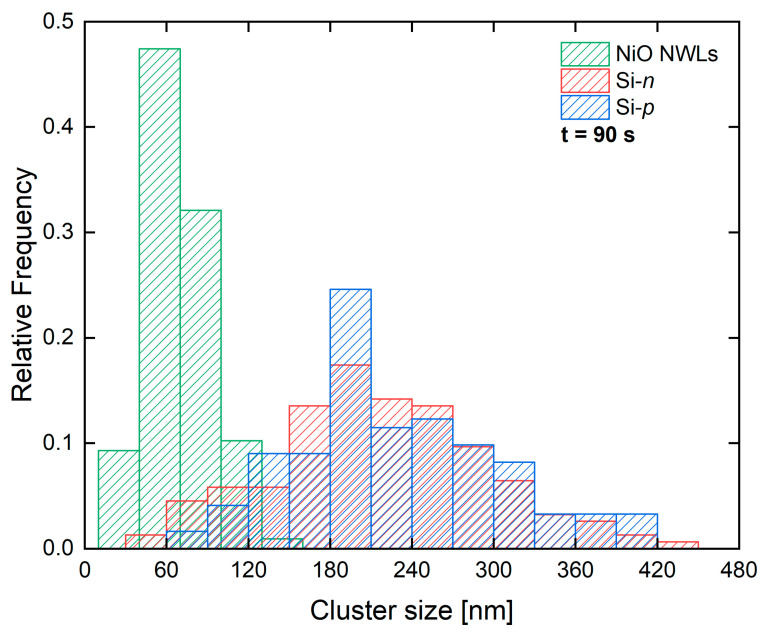
Cluster size distribution for NiO NWLs_90, Si-*n*_90 and Si-*p*_90 samples.

To deepen the substrate role, the size distribution of Au clusters obtained onto NiO NWLs_90, Si-*n*_90 and Si-*p*_90 samples is reported in Figure 2. The size of each cluster is obtained as the square roots of cluster area. The size distribution was then estimated by counting more than 150 clusters per each sample. Again, the substrate drives the mean size and width of Au clusters, leading to a smaller average size for the NWLs substrate, and a greater one for the Si substrate, regardless of its *n*- or *p*-type nature. The NiO NWLs_90 sample is decorated with Au clusters about 70 ± 30 nm in size, while the Si substrates have Au clusters of about 200 ± 100 nm in size.

**Figure 3:**
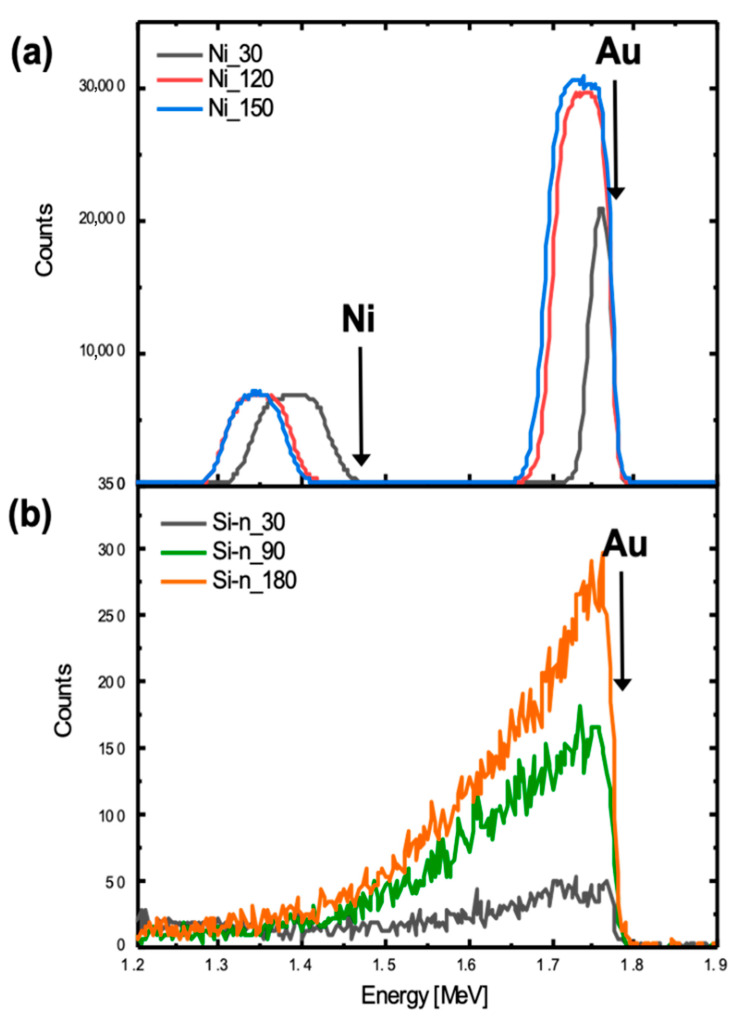
Rutherford backscattering spectrometry (RBS) spectra of (**a**) Ni samples and (**b**) Si-*n* samples after immersion in the Au solution for different immersion times (Si signal is not reported).

The effect of the substrate on ELD was investigated also by RBS analysis. Figure 3 reports RBS spectra for Ni samples (Figure 3a) and Si-*n* samples (Figure 3b) obtained for different immersion times (Si-*p* RBS spectra in Figure S3). In the RBS spectrum, a peak at 1.85 MeV or 1.47 MeV is related to He ions backscattered by Au atoms or by Ni atoms (if any) at the surface. Lower energy signals are related to backscattering events occurring below the surface. Figure 3a presents a box-like signal in the 1.7–1.85 MeV range, compatible with a Au film onto Ni [37]. The area of this box is proportional to the total Au atoms deposited onto the sample, which can be modulated by varying the ELD immersion time, as expected. The lower peak at 30 s is compatible with a Au film not fully covering the substrate. Assuming a gold density into nanoparticles as high as in bulk ($5.9\times 10^{22}$ at cm^−3^), we extracted a mean thickness of about 60 nm for 120 s of immersion time. The box at 1.3–1.45 MeV is related to Ni film, which appears shifted at lower energy when it is buried below a thicker Au film. Figure 3b presents distinct features of Au deposited onto Si-*n* (Si-*p*, significantly similar to Si-*n*, is reported in Figure S3). In comparison to the Ni substrate, the Au peak on Si-*n* is significantly lower and much broader, showing a tail at low energies. Such a feature is compatible with a 3D cluster configuration of Au agglomerates rarely spread onto the surface. Moreover, for the Si substrate, by increasing immersion time, the peaks become larger. The RBS spectra for Au deposited onto the NiO NWL and NiO substrates are reported in Figure S3. The data are in agreement with SEM analysis and add a quantitative measure of the deposited Au amount.

**Figure 4:**
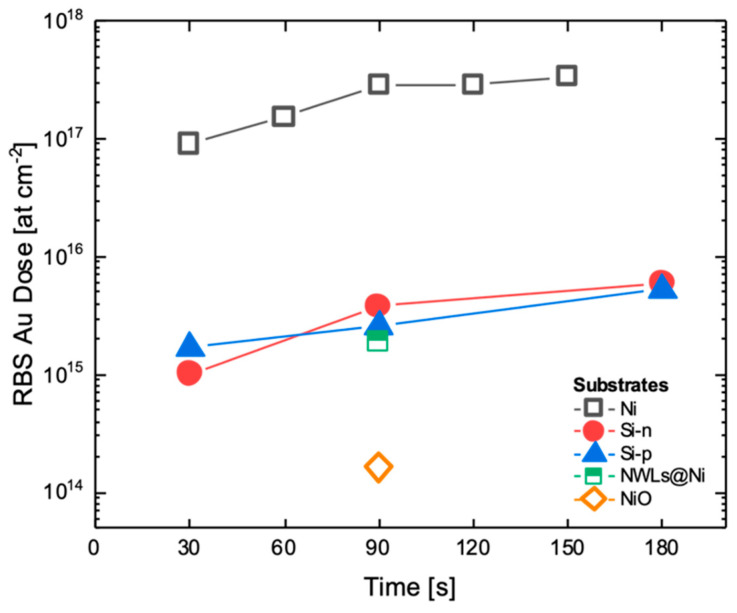
Comparison of Au dose extracted by RBS for different substrates at increasing immersion times.

The areal density of Au atoms was extracted from the integral of Au signal in the RBS spectra [36]. In order to better visualize the differences between samples, in Figure 4, the Au dose is plotted as a function of immersion time. The Au dose deposited onto the Ni substrates is at least two orders of magnitude higher than in the Si and in NiO NWLs samples. The NiO substrate induces a very weak Au deposition with a dose more than 3 orders of magnitude lower than on the Ni substrate.

For the Ni and the two Si substrates, the total amount of deposited Au is fairly linear with the time. A linear fit (with fixed intercept at zero) gives a rate of $2.5\times 10^{15}$ at cm^−2^ s^−1^ for the Ni substrate, and $3.4\times 10^{13}$ at cm^−2^ s^−1^ and $2.9\times 10^{13}$ at cm^−2^ s^−1^ for Si-*n* and Si-*p*, respectively (Figure S4). As expected, on the Ni substrate, the Au deposition proceeds with a rate 100 times faster than on the Si substrates, while the Si doping seems to be almost ineffective as far as the deposited amount of Au is concerned. This is a quantitative indication that the substrate significantly affects the Au electroless deposition process.

**Figure 5:**
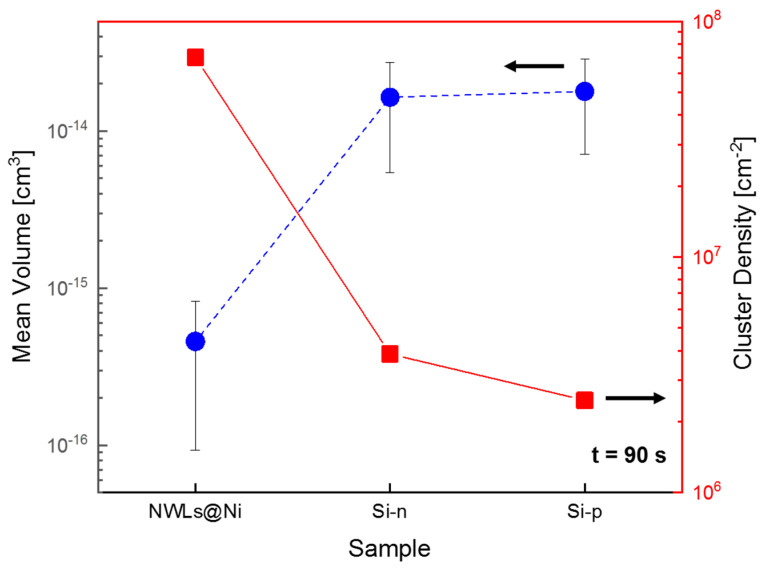
Mean volume and cluster densities for the NiO NWL_90, Si-*n*_90 and Si-*p*_90 samples.

The total amount of Au obtained by RBS can be linked with the size distributions (Figure 2) to extract the Au cluster density on the surface after 90 s. In fact, the Au dose is similar for Si-*p*_90 ($2.6\times 10^{15}$ at cm^−2^), Si-*n*_90 ($3.7\times 10^{15}$ at cm^−2^) and NiO NWLs_90 ($1.9\times 10^{15}$ at cm^−2^), however, the average size remains 3 times smaller for the last sample. Assuming a cubic shape for the clusters, the volume distribution was calculated based on the size distribution and the mean volume extracted (Figure 5). Then, by considering Au atomic density ($5.9\times 10^{22}$ at cm^−2^), the cluster density was extracted to account for the RBS Au dose (Figure 5).

The different size distribution strongly affects the mean volume values: the NiO NWLs sample presents Au clusters with a mean volume 40 times smaller than those on the Si substrates. Moreover, the same NiO NWL sample presents a cluster density 20–30 times greater than the Si substrates.

## 4. Discussion

The very same ELD process (composed of HH pre-bath followed by ELD immersion) leads to a considerably different Au deposition on different substrates, spanning from a fast deposition of a 2D film on metallic Ni to a slow process on semiconductor or nanostructured substrates, leading to 3D clusters decoration. In the following, we will discuss the Au ELD results on different substrates.

On the Ni substrate, the extracted Au deposition rate (0.42 nm s^−1^) agrees well with those present in the literature (0.64 nm s^−1^ for Swan and Gostin [16]; 0.22 nm s^−1^ for Vratny [38]) in the presence of hypophosphite, whilst it is about 4 times larger than that found by Liu (0.11 nm s^−1^) without a reducing agent [39]. Such evidence indicates a clear action of the reducing agent in improving the gold deposition rate, as found by Vorobyova [31]. Electroless deposition using Kau(CN)_2_ as a metal source and hypophosphite as a reducing agent [38,39] is shown to proceed by a local cell mechanism with a local cathode hosting Au reduction and a local anode where oxidation of substrate surface occurs [39].

**Figure 6:**
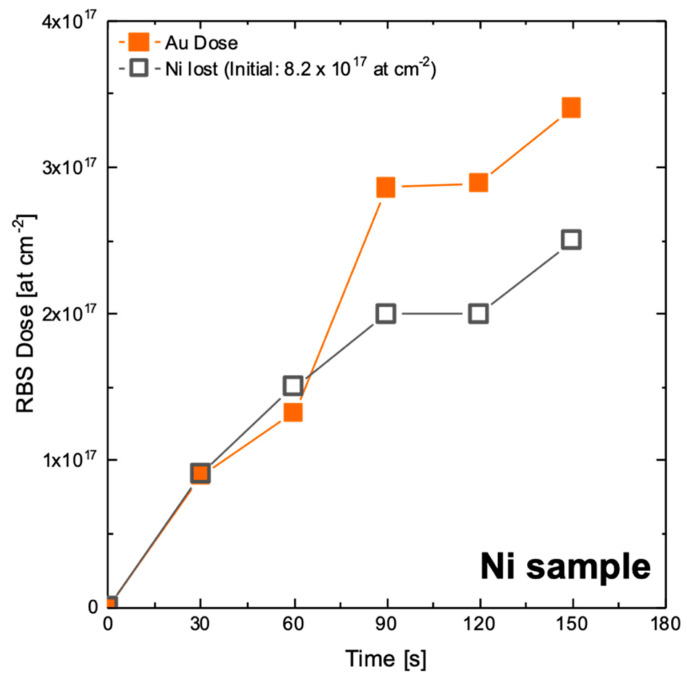
Au dose (orange closed squares) and Ni lost (grey open squares) as a function of immersion time.

Indeed, these considerations explain our results well with a Ni-Au displacement process. In fact, as ELD immersion time goes on, the Au amount increases, and the Ni amount lost by the substrate (100 nm Ni film) decreases. In Figure 6, the time variation of Au dose is compared to that of Ni lost (Figure S5 shows relative RBS spectra). Up to 60 s, there is clear evidence that for each deposited Au atom, a Ni atom from the substrate is lost. This is indicative of strong involvement of the Ni substrate in Au reduction, according to a possible reaction:

Ni + 2[Au(CN)_2_]^−^ ⟶ 2Au + Ni^2+^ + 4CN^−^ (1)

The effectiveness of the reaction is justified by the standard redox potential of the Au^+^/Au species (1.83 eV vs. SHE) [40] with respect to the position of the Fermi level of Ni (5.04 eV) [41].

For times longer than 90 s, the amount of Au slightly exceeds that of Ni lost. Such a result could support the hypothesis that beyond the displacement mechanism, a hypophosphite-mediated process should assist Au deposition. At 90 s, the average thickness of Au is around 50 nm, and the role of Ni displacement from the buried film is reasonably less effective than at early stages of ELD. It should be also noted that Liu and co-workers reported after 100–150 s a variation of the mixed potential leading to a plateau, which was attributed to the build-up of gold film on the nickel substrate [39]. Vorobyova, investigating the role of a hypophosphite-containing bath for Au ELD, found that the reducing agent does not directly affect the reduction of Au ions, but its reducing effect is mediated by the substrate depending on its composition, morphology and conductivity [31]. Thus, it is possible that a combined effect is present during the Au deposition process on the Ni substrates: in the first stage a galvanic displacement occurs, leading to high deposition rates; then, the hypophosphite-mediated process becomes predominant, with a lower deposition rate and a reduction of the dissolution of Ni of the substrate. This combined effect could maintain a fairly constant rate of the deposition process.

On the Si substrate, the Au ELD also proceeds with a local cell mechanism, through the injection of a hole in the valence band per each reduced Au atom, leading to a concomitant Si oxidation [23,42]. Nonetheless, some effects of the reducing agent mediated by the substrate cannot be discharged at all. As a matter of fact, on the Si substrate, we observe a 100 times lower rate for Au ELD than on the Ni substrate, as the conductivity of Si is 5-6 orders of magnitude lower than Ni. Indeed, the negligible difference among Si-*p* and Si-*n* can be explained by considering the surface states pinning the Fermi level of semiconductors to a midgap position and making the charge transport just below the surface almost independent of the doping [43]. Such a picture is also supported by the extremely low Au amount obtained by 90 s ELD onto NiO. In fact, the energy gap of this last semiconductor (3.6 eV) is well higher than that of Si (1.12 eV), leading to a much lower conductivity and ELD rate.

The amount of deposited Au by 90 s of ELD on NiO NWLs is comparable to that of Si, however, the NWL material remains NiO, and the substrate is not flat at all. In fact, at 90 s, the Au amount deposited on NiO NWLs is 10 times larger than on NiO. The substrate morphology must also be considered, as the extremely thin nanowalls (10–20 nm thin, [44]) can reduce the surface conductivity of NiO on the one hand, but can enhance the nucleation process on the other, because of the edge-enhanced local electric field. It is worth noting that NiO NWLs grown onto a graphene paper substrate and subjected to the same Au ELD revealed a Au dose of $3\times 10^{15}$ at cm^−2^ (the RBS spectrum is reported in Figure S6), thus excluding any relevant effect of the substrate below NWLs (Ni or graphene paper).

**Figure 7:**
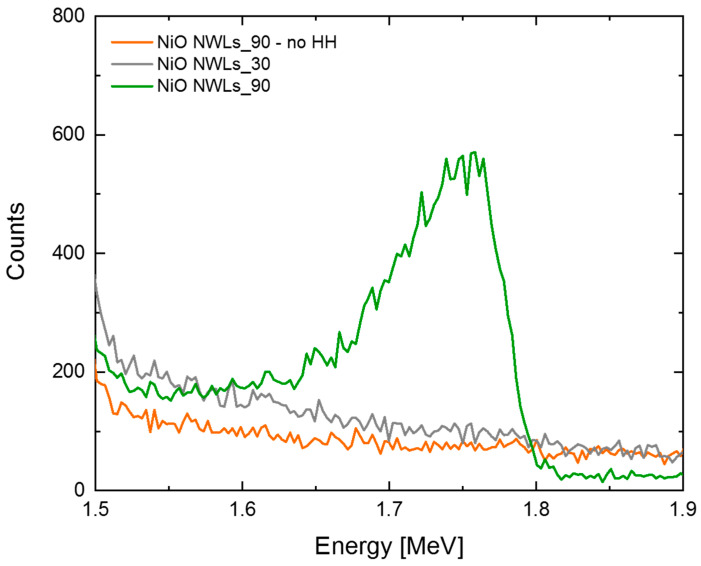
RBS spectra of NiO NWLs immersed for 30 and 90 s in the ELD solution with (NiO NWLs_30, NiO NWLs_90) and without (NiO NWLs_90-no HH) HH pre-bath.

Moreover, HH pre-bath plays a significant role. We tried to conduct the same Au ELD process without HH pre-bath (NiO NWLs_90—no HH), and RBS shows no presence of Au (Figure 7). This HH pre-bath drives a reduction of the oxidized Ni species on the substrate surface prior to ELD immersion [34], which is essential for starting Au deposition in this sample. If the ELD immersion time is reduced to 30 s, no Au is detected, even after HH pre-bath (NiO NWLs_30 in Figure 7). This datum reveals an incubation time for Au cluster deposition onto the NiO NWLs sample, differently from what is observed for the Si and Ni samples. In addition, on the NiO NWLs sample, Au cluster density is the highest ($8\times 10^{7}$ cm^−2^), and most of the clusters lie on top of the NWLs, indicating that the NWLs edge could be an effective nucleation site for Au deposition after a proper HH pre-bath.

**Figure 8:**
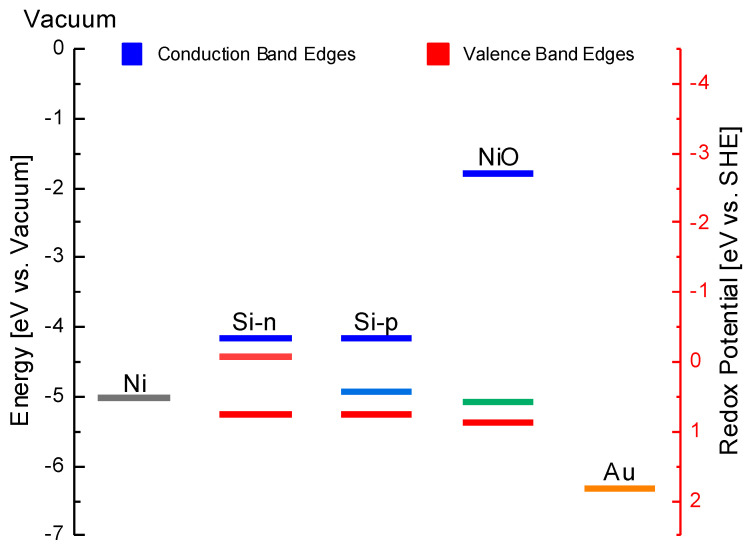
Diagram of electron energy levels on Ni, Si-*n*, Si-*p* and NiO band edges (Fermi level, conduction and valence bands) and the Au+/Au redox system in solution [40,41,45].

Given the above, we can focus our attention on the mechanism of Au cluster formation on different substrates. It is necessary to note that Au deposition (both as a 2D film or as 3D clusters) relies on transient formation of a galvanic cell at the surface where Au is reduced and Ni (or Si) oxidized, and on a hypophosphite-mediated process through a charge exchange with the substrate [22,39]. The effectiveness of these local cells depends on different factors: (i) the electron density at the sample surface, required for Au reduction and (ii) the energy position of the Au^+^/Au redox couple with respect to the Fermi level position of Ni and/or to the valence band of Si and NiO (Figure 8).

The Ni Fermi level is observed to be higher than the redox potential of Au and, therefore, allows a significant transfer of electrons through the metal–electrolyte interface.

For both Si and NiO, the valence band is still higher with respect to the redox of Au, ensuring the electron transfer through the valence band. In both Si-*n* and Si-*p* cases, the high overlap between the valence band edges and the redox level of Au+/Au explains why doping seems to not affect Au deposition.

In NiO, the intrinsic carrier density ($1.1\times 10^{6}$ cm^−3^) is much lower than in Si ($3.0\times 10^{11}$ cm^−3^) [45,46], accounting for the large difference in the amount of Au observed on the two semiconductors.

## 5. Conclusions

Au electroless deposition by using Au potassium cyanide and sodium hypophosphite has been investigated on different substrates pre-treated by a hydrazine hydrate bath. A paramount effect of substrate is demonstrated, driving the Au deposition from thin film (onto metallic substrates) to 50–200 nm clusters (on semiconducting substrates) whose size and density greatly depend on substrate material and morphology. Hydrazine hydrate bath is found to assist the nucleation process in NiO nanostructures, while the Au electroless process has been investigated. A local cell mechanism, by which a temporary anode and cathode form onto the surface, promotes the displacement process. The higher the electrical density at the surfaces, the more effective the process. The relative position of Fermi level of the metal and valence band of the semiconductor samples in respect to the redox system Au^+^/Au ensure the electron transfer between the substrate–electrolyte interface. A reproducible and controlled Au cluster decoration of NiO nanostructures can be obtained by the proposed low-cost recipe.

## Figures and Tables

**Table 1:** Summary of prepared samples.

Sample	Substrate	ELD Bath Immersion Time (s)
Ni	Ni layer	30, 60, 90, 120, 150
NiO	NiO layer	90
Si-*n*	*n*-type c-Si	30, 90, 180
Si-*p*	*p*-type c-Si	30, 90, 180
NiO NWLs	NiO nanowalls	30, 90

## Supplementary Materials

The following are available online at https://www.mdpi.com/2079-4991/10/11/2180/s1, Figure S1. EDX spectrum for Ni sample obtained by Au ELD for 90 s. Presence of Ni and Au is indicated by labels on respective peaks; Figure S2. Plan-view SEM images of Si-*p* sample obtained by Au ELD for 90 s; Figure S3. RBS spectra of (a) Si-*p* samples after immersion in ELD solution for different immersion times (Si signal is not reported), (b) NiO NWLs_90 and (c) NiO_90. The inset in (c) shows Au peak in the region between 1.6 MeV and 1.9 MeV; Figure S4. Linear fit of Au RBS doses for (a) Ni, (b) Si-*n* and Si-*p* sample. The intercept was fixed to zero in all cases, while slopes represent the rates of deposition of Au; Figure S5. RBS of Ni sample: (a) Ni and (b) Au peaks at different immersion time (from 30 s to 150 s); Figure 6. RBS spectrum of Au deposited by ELD on NiO NWLs grown on Graphene Paper (GP).
