# Supplementary material for: Role of Substrate in Au Nanoparticle Decoration by Electroless Deposition
Source: Nanomaterials (Basel). 2020 Nov 1;10(11):2180. doi: 10.3390/nano10112180 (PMC7692156; doi:10.3390/nano10112180)
Supplement: Supplementary file 1 [file nanomaterials-10-02180-s001.pdf]

Role of Substrate in Au Nanoparticle Decoration by Electroless Deposition

Luca Bruno [1,*], Mario Urso [1], Yosi Shacham-Diamand [2], Francesco Priolo [1] and Salvo Mirabella [1]

[1] Dipartimento di Fisica e Astronomia "Ettore Majorana", Università di Catania, and IMM-CNR, via S. Sofia 64, 95123 Catania, Italy; mario.urso@dfa.unict.it (M.U.); francesco.priolo@ct.infn.it (F.P.); salvo.mirabella@dfa.unict.it (S.M.)

[2] Department of Physical Electronics, School of Electrical Engineering and Department of Materials Science and Engineering, Faculty of Engineering, Tel Aviv University, Tel-Aviv 69978, Israel; YosiSh@tauex.tau.ac.il

* Correspondence: luca.bruno@dfa.unict.it

Received: 24 September 2020; Accepted: 29 October 2020; Published: date

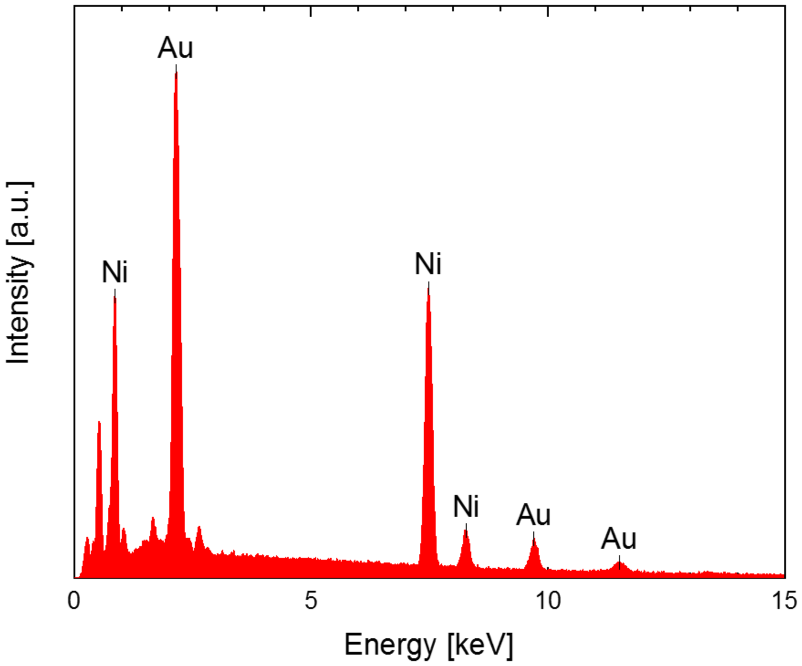

Figure S1. EDX spectrum for Ni sample obtained by Au ELD for 90 s. Presence of Ni and Au is indicated by labels on respective peaks.

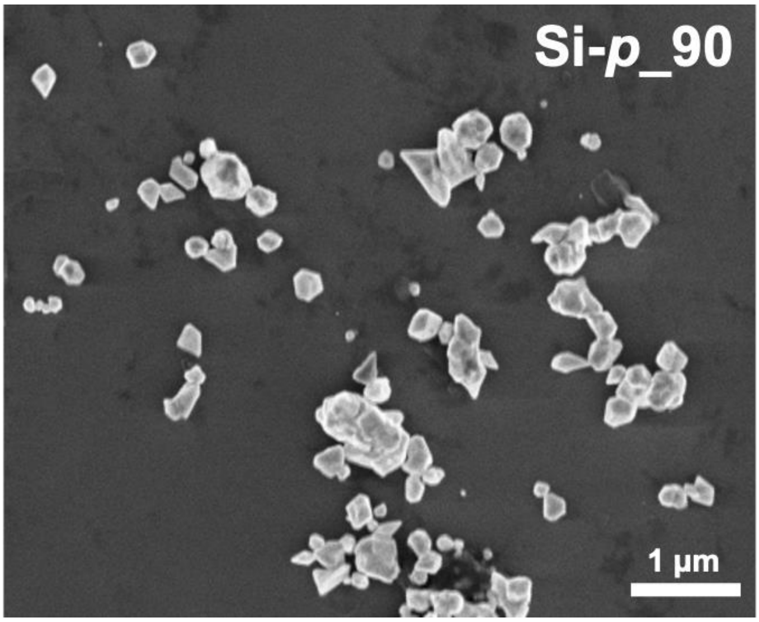

Figure S2. Plan-view SEM images of Si-*p* sample obtained by Au ELD for 90 s.

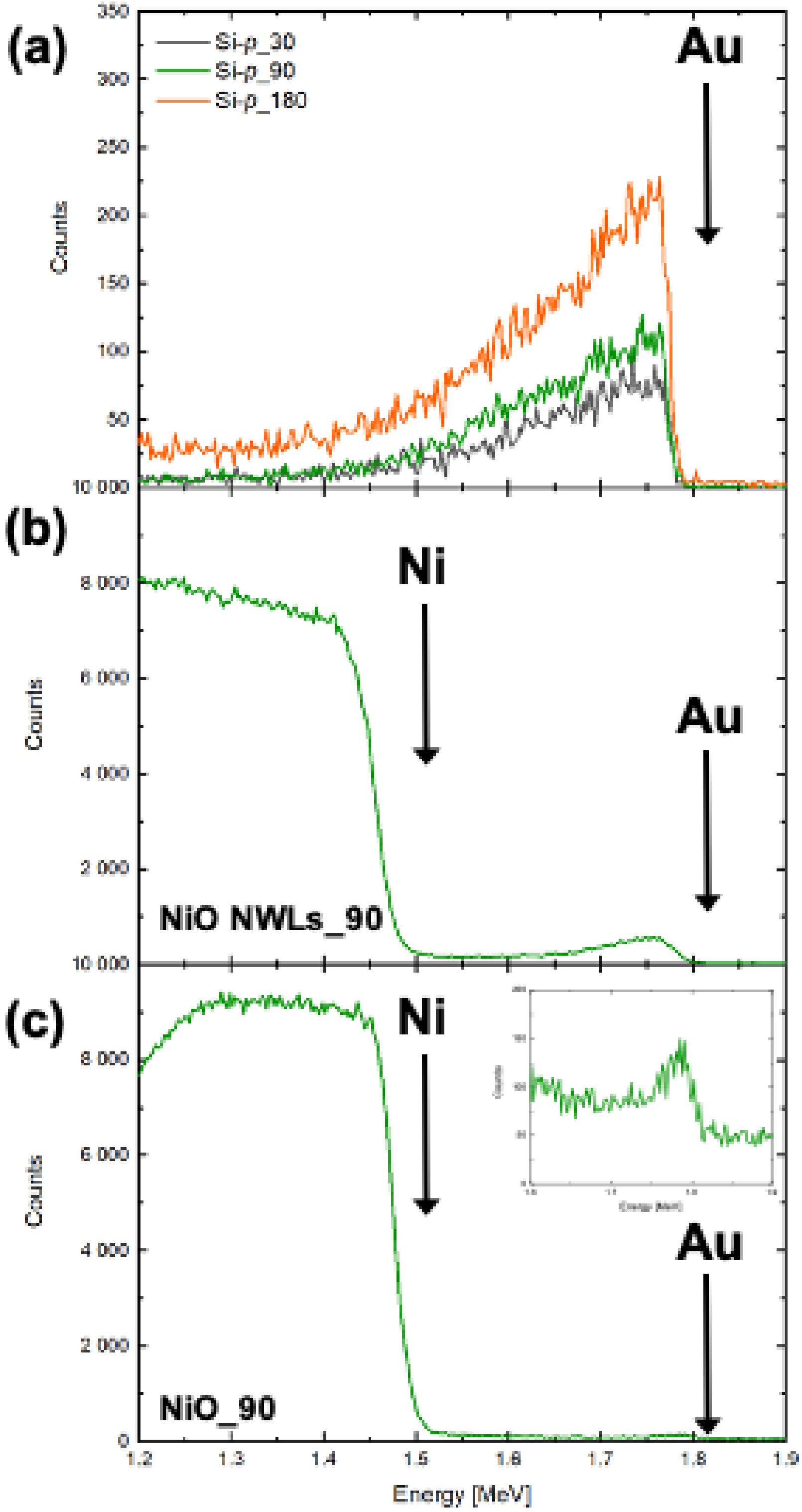

Figure S3. RBS spectra of (a) Si-*p* samples after immersion in ELD solution for different immersion times (Si signal is not reported), (b) NiO NWLs_90 and (c) NiO_90. The inset in (c) shows Au peak in the region between 1.6 MeV and 1.9 MeV.

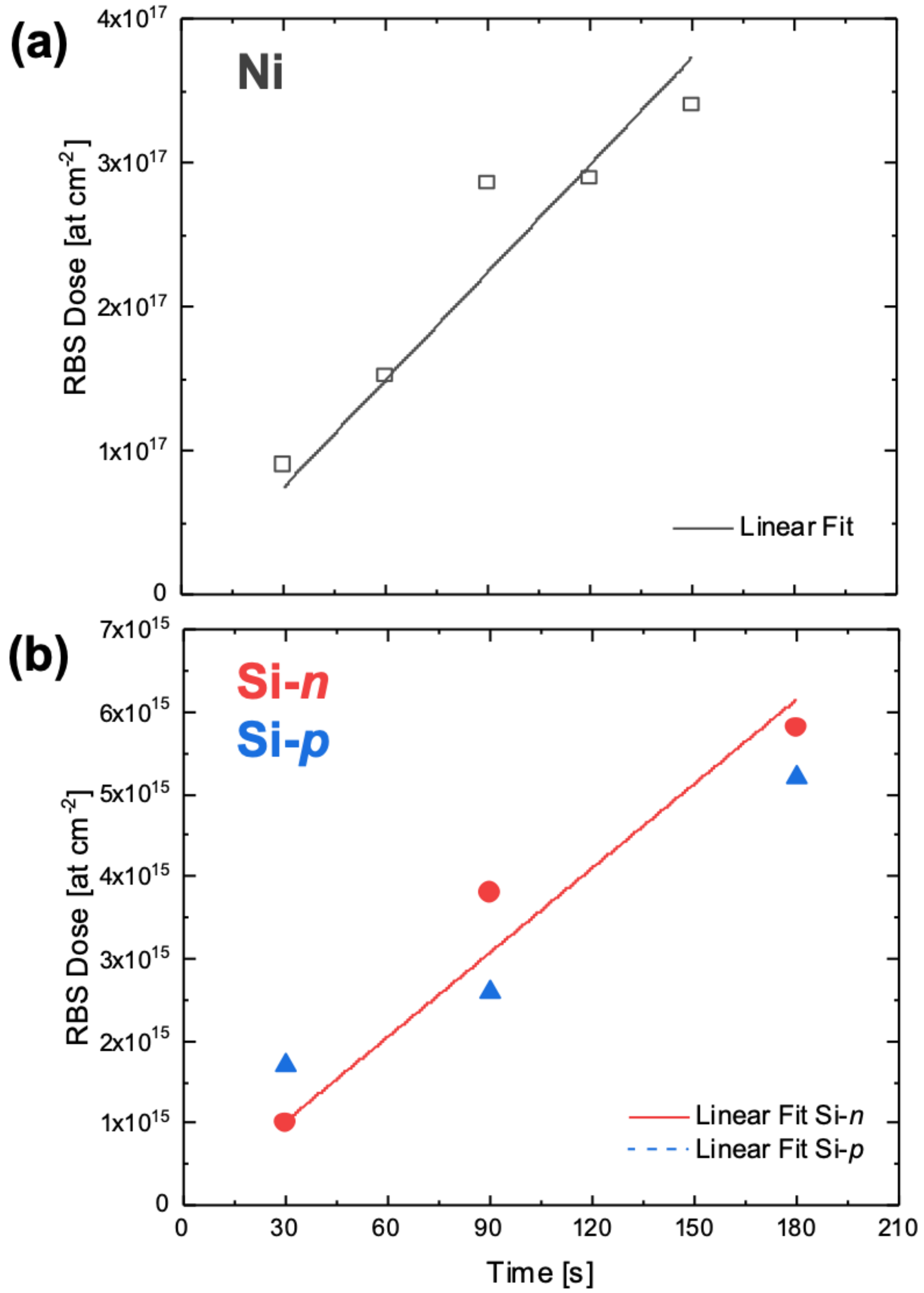

Figure S4. Linear fit of Au RBS doses for (a) Ni, (b) Si-*n* and Si-*p* sample. The intercept was fixed to zero in all cases, while slopes represent the rates of deposition of Au.

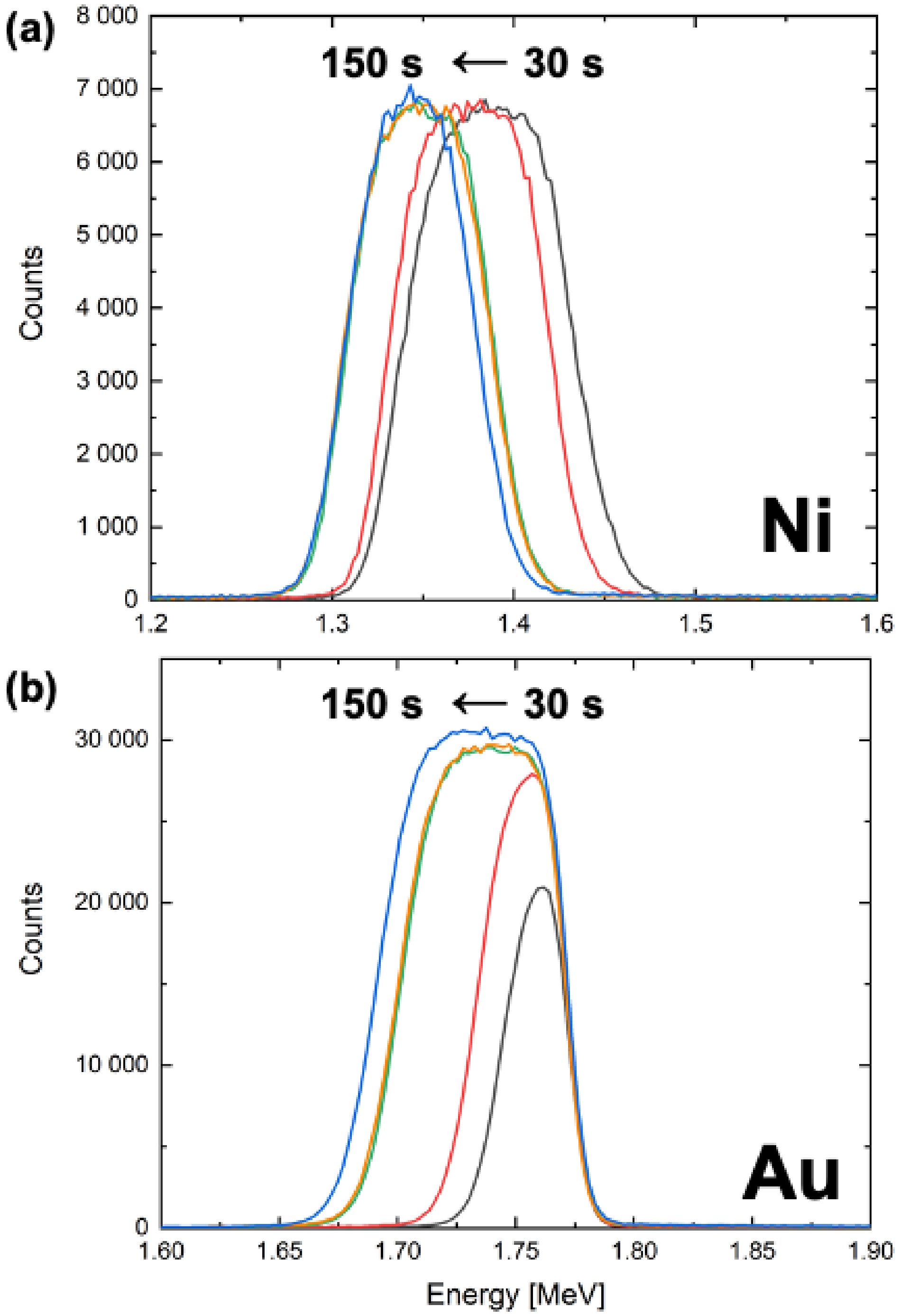

Figure S5. RBS of Ni sample: (a) Ni and (b) Au peaks at different immersion time (from 30 s to 150 s).

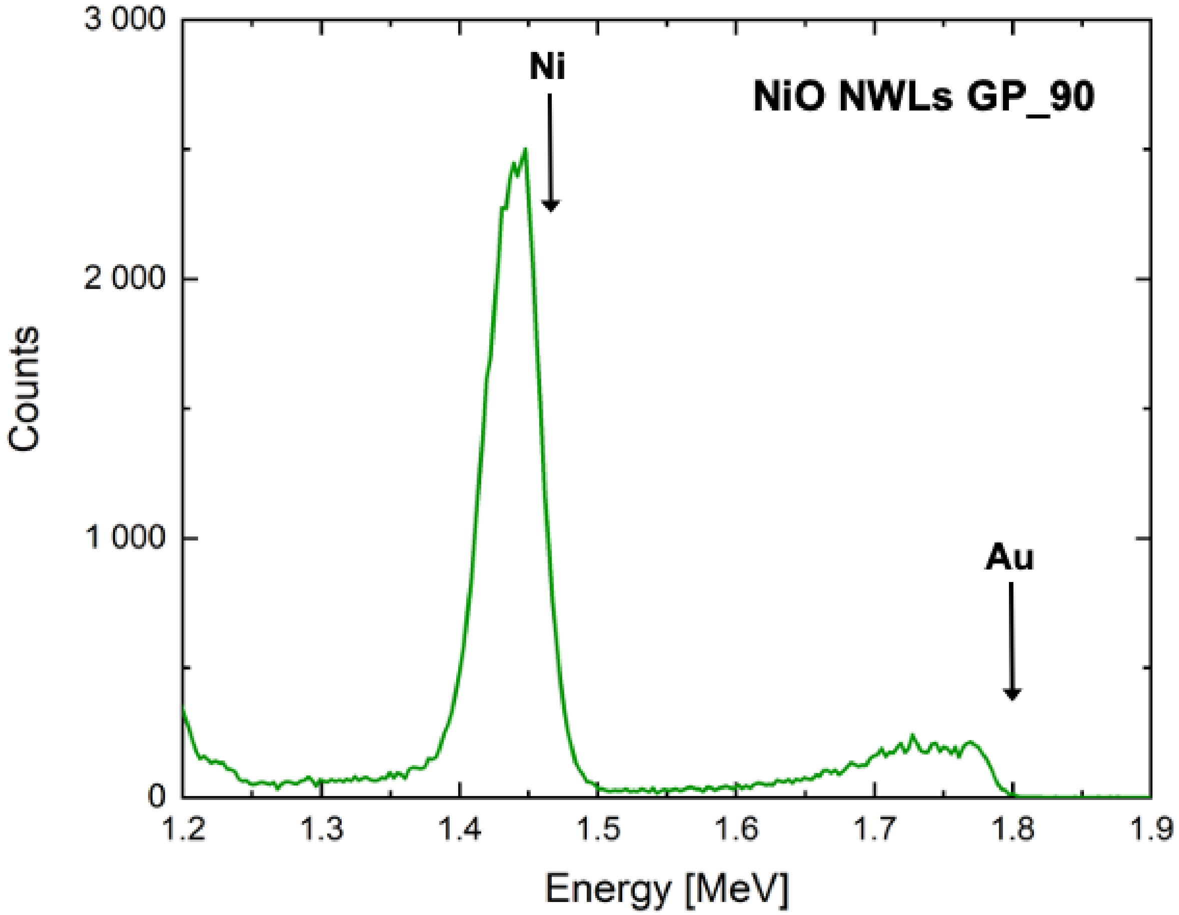

Figure 6. RBS spectrum of Au deposited by ELD on NiO NWLs grown on Graphene Paper (GP).
